# Progress of Single-Cell RNA Sequencing Technology in Myocardial Infarction Research

**DOI:** 10.3389/fcvm.2022.768834

**Published:** 2022-02-17

**Authors:** Lanfang Li, Min Wang, Qiuxiao Ma, Yunxiu Li, Jingxue Ye, Xiaobo Sun, Guibo Sun

**Affiliations:** ^1^Institute of Medicinal Plant Development, Peking Union Medical College and Chinese Academy of Medical Sciences, Beijing, China; ^2^Xiyuan Hospital, China Academy of Chinese Medical Sciences, Beijing, China; ^3^Key Laboratory of Natural Medicines of the Changbai Mountain, Ministry of Education, Molecular Medicine Research Centre, College of Integration Science, College of Pharmacy, Yanbian University, Yanji, China

**Keywords:** angiogenesis, cardiomyocyte, fibroblast, single-cell RNA sequencing, myocardial infarction, immune cell

## Abstract

After myocardial infarction, the heart enters a remodeling and repair phase that involves myocardial cell damage, inflammatory response, fibroblast activation, and, ultimately, angiogenesis. In this process, the proportions and functions of cardiomyocytes, immune cells, fibroblasts, endothelial cells, and other cells change. Identification of the potential differences in gene expression among cell types and/or transcriptome heterogeneity among cells of the same type greatly contribute to understanding the cellular changes that occur in heart and disease conditions. Recent advent of the single-cell transcriptome sequencing technology has facilitated the exploration of single cell diversity as well as comprehensive elucidation of the natural history and molecular mechanisms of myocardial infarction. In this manner, novel putative therapeutic targets for myocardial infarction treatment may be detected and clinically applied.

## Introduction

**Figure 1:**
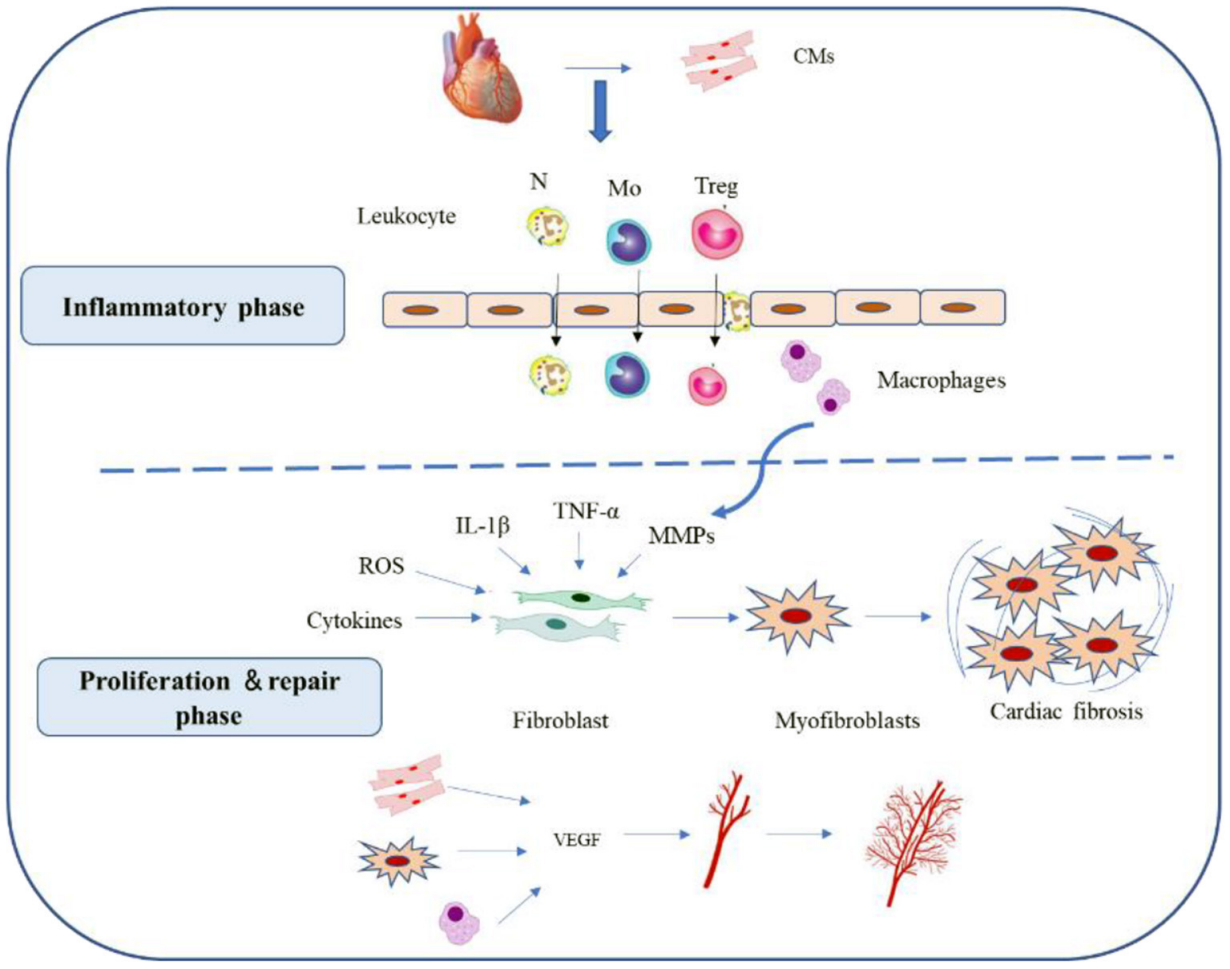
Reparative response following a myocardial infarction. Cardiac repair after MI results from a finely orchestrated and complex series of events, initiated by intense sterile inflammation, and immune cell infiltration (inflammatory phase) that serve to digest and clear damaged cells and extracellular matrix tissue, followed by a proliferation and repair phase with resolution of inflammation, (myo)fibroblast proliferation, scar formation, and angiogenesis over the next several days. CMs, cardiomyocytes; N, neutrophil; MO, monocytes; Treg, Regulatory cells; ROS, reactive oxygen species; VEGF, vascular endothelial growth factor.

Myocardial infarction (MI) is a common cardiovascular disease. It is expected to become the leading cause of death worldwide in the near future (1). The underlying pathogenesis of MI is primarily coronary atherosclerosis (2, 3). MI progression involves complex interactions among cardiomyocytes, non-cardiomyocytes, and other cell types (4), including fibroblast activation, immune cell infiltration, angiogenesis. MI repair comprises the inflammatory, proliferative, and mature stages (5) (Figure 1). Identifying the changes in myocardial cell function, transcription factors, and the molecular pathways associated with the foregoing cellular processes may facilitate the development of novel therapeutic strategies (5, 6). The multifactorial nature of MI renders study design and interpretation very difficult. By contrast, it also provides multiple therapeutic targets. Hence, a comprehensive understanding of MI pathology is necessary before specific interventions can be applied. It may be achieved *via* cell-specific assessments of the genetic and molecular mechanisms that drive MI progression.

Genome-wide transcriptome analyses have greatly advanced our understanding of the underlying biology and the regulatory networks that drive cardiac disease mechanisms (7, 8). However, studies of cardiac gene expression have been limited to the whole-tissue level. Tissue homogenates may lose information regarding cell origins and cell-specific changes in gene expression. Thus, single-cell sequencing came. So what is single-cell sequencing? As the name implies, single-cell sequencing is the sequencing technology to obtain the genetic information of a single cell. So why use single-cell sequencing? For multicellular organisms, there are differences from cell to cell. Traditional methods of research are carried out at the multicellular level. For example, bulk RNA sequencing. In addition, the advent of single-cell sequencing has paved the way for comprehensive cardiac biology studies that do not rely on cell surface or genetic pedigree tracking markers (9). These markers are often imperfect and might be heterogeneous even in populations with similar cell types (6). The outstanding advantage of single-cell sequencing technology is that it can detect the cell specificity and differences between cells, explore the cooperative operation mode between cells and study the problem of tissue heterogeneity from the perspective of cell atlas. In-depth characterization of diseases based on cellular features. Then combined with multi-omics analysis, cell and molecular imaging and other technologies, more accurate cell maps can be drawn to deepen the understanding of the law of disease evolution, and provide powerful help for finding new targets for disease treatment and exploring the cell development process. Currently, the scRNA-seq technique is used primarily to disclose cellular heterogeneity, identify novel marker genes and cell subsets, identifying rare cardiac cell types, inferring the trajectory tree, estimating RNA velocity, elucidating the cell–cell communication, and comparing healthy and pathological heart samples, and so on (10).

**Figure 2:**
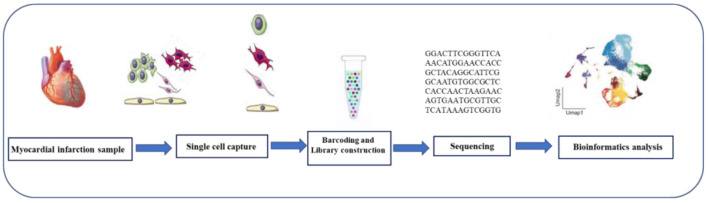
Single-cell RNA sequencing workflow. The scRNA-seq process includes tissue digestion, isolation of single cell or single nucleus, reverse transcription, cDNA amplification, construction of sequencing libraries, sequencing, and data analysis.

Single cell sequencing mainly includes single cell genome sequencing, single cell epigenome sequencing, single cell transcriptome sequencing (scRNA-seq). Several different scRNA-seq methods have been developed, such as SMART-seq, Drop-seq, 10X Genomics, and so on. Most of them have the same workflow, namely, single cell isolation and capture, cell lysis, RNA reverse transcription, cDNA amplification, library preparation, sequencing, and data analysis (11) (Figure 2). Numerous computational tools have been developed in parallel with the development of single-cell sequencing methods. For example, pseudo time analysis, sequencing single cells along the trajectory according to the similarity of expression patterns among sequenced cells to simulate the process of cell dynamic change, is a common and important method in single cell research and application (12). Pedigree relationships are revealed experimentally by the fate mapping method, which is called pedigree tracing (also known as pedigree tracing) when it is performed at single-cell resolution (13). Lineage tracing offers a powerful means of understanding tissue development, homeostasis, and disease. Single-cell transcriptomics allows the study of the transcriptional state of thousands of individual cells to reliably analyze the diversity of cell types and transitions between different states in heterogeneous samples, combined with lineage tracing to accurately predict tissue construction or maintenance of differentiation trajectories.

In this review, we examined the application of single-cell sequencing in MI to clarify the main biological phenomena occurring during this disease process (Table 1). We also described the major limitations of this tool and discussed prospects for its future application in cardiovascular research.

**Table 1:** Application of single cell sequencing technology in the study of myocardial infarction.

**Publication**	**Publication year**	**Species/tissue**	**Age**	**Cell type**	**Model**	**Technique**
Kanisicak et al. (14)	2016	Mouse/ventricles	8w	Non-CMs	1 week after MI	Fluidigm C1
Gladka et al. (15)	2021	Mouse/infarcted area	8–9w	CMs	3,14,28 days after MI	SORT-seq
Tombor et al. (16)	2021	Mouse/heart	10–12w	ECs	3–7 days after MI	10X Genomics
Farbehi et al. (17)	2019	Mouse/heart	8–12w	Non-CMs	days 3 and 7 post- MI	10X Genomics
Kretzschmar et al. (18)	2018	Mouse/ ventricles	1w; 8w	CMs and non-CMs	14 days after MI	CEL-Seq2
Ruiz-Villalba et al. (19)	2020	Mouse/ ventricles	8–10w	CFs	7, 14, 30 days after MI	10X Genomics
Forte et al. (20)	2020	Mouse/ ventricles	10–12w	non-CMs	1, 3, 5, 7, 14, and 28 days after MI	10X Genomics
Wang et al. (21)	2020	Mouse/ ventricles	P1; P8	CM and non-CMs	1 and 3 days after MI	10X Genomics
Zhuang et al. (22)	2020	Mouse/heart	8–10w	CMs and non-CMs	3 and 7 days after MI	–
Li et al. (23)	2019	Mouse/heart	8–10w	ECs	7 days after MI	10X Genomics
Cui et al. (24)	2020	Mouse/infarcted area	P2; P4; P9; P11	CMs	1 and 3 days after MI	10X Genomics
Heinrichs et al. (25)	2021	Mouse/heart and mediastinal lymph nodes	8–9w	B cells	1, 3, 7and 14, days after MI	10X Genomics

*CMs, cardiomyocytes; ECs, endothelial cells; CFs, Cardiac fibroblasts MI; myocardial infarction; w, week; P, postnatal day; “–”, data from integrated three representative datasets*.

## Myocardial Cell Damage

When MI occurs, persistent hypoxia and ATP deficiency in myocardial cells cause activation of the apoptotic cascade and myocardial cell necrosis. As CMs have a poor regeneration ability, many of them are lost, and ventricular pathological remodeling, significantly impaired cardiac function, and eventual heart failure may follow. Currently available treatments do not directly replace the cardiomyocytes lost because of heart damage and long-term post-MI prognosis is poor. However, more recently, a few studies (26, 27) induced post-MI cardiomyocyte cycle reentry to promote cardiomyocyte proliferation, and identified several candidate factors that control cardiomyocyte proliferation, such as cell cycle regulators [cyclin or its dependent kinases (28), myeloid ecotropic viral integration site 1 homolog (MEIS1) (29)], pathophysiological factors [oxidant stress (30), energy metabolism (30), ECM microenvironment (31)], and so on. Single-cell sequencing of the heart has provided new insights into cardiomyocyte cycle reentry.

Microfluid-based single-cell RNA-seq is not feasible as cardiomyocytes are large and cardiomyocytes have more than one nucleus. Thus, most studies performed single-nucleus sequencing (snRNA-seq) on cardiomyocytes (32). Although snRNA-seq offers a viable alternative to scRNA-seq for the identification of cell types in tissue for which cell dissociation is problematic, it also reduces transcript detection sensitivity and results in loss of isoform-specific information, such as cellular states (33) and active translation mRNA that defines the biological activity of cardiomyocytes. In addition, any droplet based scRNA-seq approach would lose information about the number of cardiomyocyte nuclei and cell mass. Yekelchyk et al. (32) describe a novel method, based on the ICELL8 single-cell system, for single-cell RNA-sequencing of intact mono- and multi-nucleated cardiomyocyte. They reported that adult cardiomyocytes were homogenous, mononuclear, binuclear, or multinucleate and expressed nearly identical gene sets. Hypertrophic cardiomyocytes underwent significant transcriptional changes and were highly heterogeneous. To develop a reliable gene expression profile for all cardiomyocyte types, Gladka et al. (34) first identified optimal tissue digestion and RNA extraction methods for high quality RNA extraction from single cell suspensions of the adult heart. Based on cell imaging and assessment of RNA quality by RNA integrity index (35), digestive juices containing free enzymes are ideal for dispersing adult heart tissue while preserving intact RNA.

Using mononuclear RNA sequencing of cardiomyocytes from both damaged and healthy adult hearts, See et al. (36) discussed the CMs from failing and non-failing mammalian hearts, revealing the heterogeneity of the *in vivo* myocardial stress-gene response. The study noted distinct sub-populations of CMs and uncovered gene regulatory networks specific for each sub-population, displaying specific sub-group upregulation of cell cycle, and de-differentiation genes in the disease stress response. The study identified long intergenic non-coding RNAs (lincRNAs) as key node regulators in the cardiomyocyte cycle (36), which indicates that adult cardiomyocyte subsets may have the endogenous potential for *in vivo* cardiac regeneration. The neonatal heart can readily regenerate within a short period after birth (37). Wang et al. (21) performed single-cell RNA sequencing on neonatal hearts at various time points following myocardial infarction, identified CCL24, a macrophage secreted factor specifically expressed after P1 MI, which can promote CM proliferation. Zhang et al. (38) used snRNA-seq to study cardiomyocytes and revealed that the observed increase in endogenous cardiomyocyte renewal in post-MI hearts was caused by the dedifferentiation and proliferation of pre-existing cardiomyocytes rather than the differentiation of adult cardiac progenitor cells. Honkoop et al. (39) used single-cell analysis identified a group of heart muscle cells close to the site of the wound that multiplied to repair the damage, and uncovered that metabolic reprogramming by ErbB2 signaling is essential for cardiomyocyte proliferation in the regenerating heart. Cui et al. (24) used anti-PCM1 (pericentriolar material 1) antibodies to perform snRNA-seq on healthy and infarcted myocardial tissue samples. This study describes the dynamic transcriptional landscape of different cardiomyocytes in newborn mice. Immature cardiocytes, entering the cell cycle after injury, were found to exhibit a unique transcriptional program dependent on the nuclear transcription factor Y subunit α (NFYa) and the nuclear factor redline 2-like 1 (NFE2L1) transcription factors for proliferative and protective functions, respectively. Cardiac overexpression of these two factors provides protection against ischemic injury to the mature mouse heart. These data provide mechanical insights into the molecular basis of neonatal cardiac regeneration with single-cell resolution and raise the possibility that it can be manipulated to facilitate cardiac repair after injury. Studying the innate response of regenerated cardiomyocytes to injury can identify factors that can be used to improve cardiac repair and function.

The study of the structure of an organism helps uncover specific functions. Defining regions, age, disease specificity, and gene expression signatures in various cell populations may enable the identification of novel molecular mechanisms associated with heart disease and therapeutic regimens. Wang et al. (40) have analyzed ~500,000 single cells and nuclei from six different cardiac regions has expanded the adult cardiac cell map and highlighted heart cavity-specific differences between males and females. They uncovered unanticipated CMs diversity in both the left atrium (LA)3 and left ventricle (LV), characterized by preferentially expressed cell surface markers, secretory proteins, cytoskeletal genes and transcription factors in each subtype, and LA CMs displayed a more diversified molecular signature, ranging from canonical contraction and metabolism to immune regulation and muscle organ development.

Single-cell sequencing should be implemented in future studies to develop reliable strategies for generating new myocytes and to clarify if/when new myocytes are generated. More accurate identification of dividing muscle cells and a more accurate timeline of their cell cycle activity will help find the best way to induce myocardial cell renewal.

## Inflammatory Cell Infiltration

In response to myocardial ischemia injury, damaged cardiomyocytes undergo apoptosis, secrete cytokines and soluble factors, trigger inflammatory infiltration, and activate fibroblasts (41). After MI, inflammation and its resolution play key roles in promoting healing and limiting the severity of both acute cardiac damage and adverse cardiac remodeling (26, 42). As there is a strong association between inflammation and adverse outcome, proinflammatory immune cells are potential therapeutic targets. The inflammatory response in MI involves both resident heart cells and newly recruited cells. However, the relative contribution of each cell type remains unclear.

MI triggers an acute inflammatory response that causes neutrophils to move rapidly to the site of injury. But the complete functional diversity and temporal dynamics of cardiac neutrophils were incomplete. Vafadarnejad et al. (43) found temporal changes in the presence of different neutrophil states in the infarcted heart and circulatory system of mice. Neutrophils are considered transient first responders to tissue injury such as myocardial infarction. The SiglecF (mouse sialic acid-binding immunoglobulin-like lectin F) is an eosinophil surface receptor. They followed different aging trajectories and found tissue-restricted SiglecF^hi^ status. Recently, Calcagno et al. (44) also found the same phenomenon using single-cell transcriptomics, proving that SiglecF^hi^ neutrophils appeared in the late stage.

Macrophages are pluripotent cells of the innate immune system. They are indispensable in both the initial inflammatory response and subsequent cardiac wound healing. They are the most abundant immune cells in the heart and undergo significant cell flux in injured hearts (45). Zhuang et al. (22) identified seven macrophage subsets by integrating 27,349 non-myocardial cells after MI and performing single-cell sequencing. Tissue-resident macrophages were selected for the subsequent analyses, and they expressed the repair genes CD163 and Mrc1. This indicates that these cells regulate anti-inflammatory repair rather than the pro-inflammatory response during ischemic injury. Another study showed that selective depletion of tissue-hosted CCR2^−^ or CCR2^+^ macrophages during MI had different effects on left ventricular function, myocardial remodeling, and monocyte recruitment (46). Tissue-resident CCR2^+^ macrophages promoted monocyte recruitment, monocyte chemotactic protein (MCP) release, and monocyte mobilization through a bone marrow differentiation primary response 88 (MYD88)-dependent mechanism. By contrast, tissue-resident CCR2^−^ macrophages inhibited monocyte recruitment. In the present study, genotypic comparative analysis revealed significant changes in the macrophage subpopulation after MI reperfusion, which confirmed *via* immunostaining analysis. Ni et al. (47) studied cardiac macrophage (CMϕ) heterogeneity using single-cell sequencing and identified macrophage subsets associated with cardiac injury. CD72^hi^ CMϕs was identified as a proinflammatory macrophage subset. Pseudo-time trace and CHiP-seq analysis identified Rel as the key transcription factor inducing CD72^hi^ CMϕ differentiation. Bone marrow-derived, Rel-mediated CD72^hi^ macrophages play a proinflammatory role in heart injury induction and are, therefore, potential therapeutic targets for various cardiovascular diseases. Zhuang et al. found through single-cell sequencing that macrophages from various sources have different transcriptional characteristics, pathways, developmental tracks, and transcriptional regulators (22). Using single-cell sequencing, Ren et al. (48) found that macrophage activation and subtype conversion are key events in cardiac hypertrophy metaphase. Current data indicate that macrophage development, phenotype, and function are markedly heterogeneous and plastic. New insights into macrophage biology may explain the failure of non-specific immunosuppression strategies and provide new opportunities for targeted MI prophylaxis. Therefore, it is important to understand the flow of macrophages and their relative functional differences under homeostasis and ischemia.

Another study showed that lymphocytes readily infiltrate the heart after MI, and single-cell sequencing revealed that lympho B cells promote TGF-1β production in locally damaged hearts (25). Xia et al. monitored the dynamic recruitment of regulatory T cells (Tregs) in the damaged heart of a mouse MI model (49). Studies reported that Tregs are highly enriched in post-MI hearts. Furthermore, it was found that Sparc upregulation in cardiac Tregs protects the heart from MI by increasing the collagen content and promoting collagen maturation in the infarct area (49).

In addition, after myocardial infarction, the monocyte population expands at the infarct site and changes their phenotype in the mouse heart (50). Recent research has highlighted heterogeneity within classical and non-classical human monocytes, challenging the current three subset paradigm (51). Many recent preclinical studies have also focused on targeting the CCL2/CCR2 axis to alter monocyte response or inhibiting monocyte trained immunity modulate post-MI inflammation (52). Abplanalp et al. (53) demonstrated that the relative monocyte/T lymphocyte ratios were elevated in MI patients. They also reported that recombinant human fatty acid binding protein 5 (FABP5) was upregulated in the typical monocytes of patients with heart failure. Moreover, FABP5 might induce a proinflammatory monocyte phenotype in other inflammatory disease models. Therefore, FABP5 upregulation may contribute to deep inflammatory signaling induction in the typical monocytes of patients with heart failure (53). However, additional studies are needed to fully characterize the epigenetic characteristics of trained immune cells and the functional relevance of these subgroups in patients with cardiovascular disease before this can be translated into a viable cardioprotective therapy (54). In this area of research, single-cell sequencing can provide favorable conditions.

The mass cell death that occurs during myocardial infarction releases self-DNA and triggers an interferon response. King et al. (55) processed single-cell RNA sequencing analysis of white blood cells from the hearts of infarcted and non-infarcted mice showed that ischemic cell death intensifies the fatal response to myocardial infarction by activating interferon regulatory factor 3 (IRF3) and type I interferons (IFNs) production. This suggests that interference with or antibody blocking of IFNs receptors will improve function and survival of infarcted hearts. Collectively, molecular- and cell-based approaches toward modulating post-MI immune responses have emerged as promising therapeutic strategies.

Although there is compelling evidence that monocytes and macrophages play a key role in ventricular remodeling after MI, targeted immunotherapy strategies have been slow to emerge, and even strategies targeting specific subpopulations seem to produce conflicting results. This may still be related to the narrow traditional understanding and definition of cell subsets. There is still a long way to go before immunotherapy is applied to the treatment and prognosis of myocardial infarction.

## Fibroblast Activation

Cardiac fibroblast activation leads to fibrosis, maladaptive remodeling, and heart failure progression. Myofibroblasts are in the classical activation state and secrete fibrous collagen and other extracellular matrix (ECM) proteins. Therefore, they maintain the structural integrity of the compartment. Abnormal, persistent fibroblast activation contributes to heart failure progression. Recent advances in this field have shown that various fibroblast subsets occur in diseased tissue and undergo temporal variation at the time of injury (19, 22). Fibroblast states are highly complex; hence, dynamic state-space models may more accurately describe fibroblast trans-differentiation than traditional models. Targeting cardiac fibroblast activation is a novel approach toward reducing interstitial fibrosis and ameliorating MI, and single-cell sequencing can reassess these changes.

Transcriptional-level scRNA-seq analysis demonstrated the presence of a new fibroblast subpopulation as early as 1 d after MI which expressed high levels of the monocyte macrophage chemokines and the neutrophil activators (41). This indicates that this early fibroblast state is associated with inflammatory response initiation. Cell trajectory analysis revealed that these cells rapidly differentiated into myofibroblasts by day 3 after MI. Thus, fibroblasts have transcriptional plasticity and can rapidly alter the cellular landscape of damaged myocardia. In addition, the study myofibroblasts and endothelial cells are central hubs for cell communication mediated by ligand-receptor interactions. Zhuang et al. (22) found that myofibroblasts significantly increased by 7 d after MI. They promoted repair and expressed Postn, Cthrc1, and Ddah1. Nevertheless, only Ddah1 was expressed in activated fibroblasts. Farbehi et al. (17) also identified new resting and activated fibroblast states after MI which were divided into two main groups differentiated by their relative surface-labeled spinocerebellar ataxia 1 (Sca1) expression levels (17). A key finding of the scRNA-seq analysis was that the myofibroblasts were heterogeneous and continuously changed phenotype. Some became proliferating cells, while others presented with different expression levels of TGF-β1, Cilp, Thbs4, and Postn (17).

ScRNA-seq also characterizes rare cell populations that batch sequencing methods often overlook. Identifying rare cells is essential for understanding the biology of normal and diseased tissues. Ruiz-Villalba et al. identified a unique subset of cardiac fibroblasts expressing high CTHRC1 (collagen triple helix repeat containing 1) levels after MI. This process reduced the risk of death from cardiac rupture (19). In later validation test discovered thatcthrc1-deficient mouse models presented with increased mortality and reduced fibrotic response after MI.

A novel therapeutic heart failure paradigm is the successful pathogenic fibroblast targeting for disease amelioration in preclinical models (41). However, fibroblasts have widespread distribution and lack specific markers. Hence, the inability to achieve therapeutic specificity in targeting fibroblasts or other heart cell types is a major obstacle in drug development. In this manner, studies have shown that the Ckap4 marker was specifically induced in activated fibroblasts (34), and exclusive Ddah1 expression in myofibroblasts during MI healing (22) which may indicate novel activated fibroblast markers. Recently, Shi et al. (56) found the expression levels of FTO (fat mass and obesity-associated protein), YTHDF3 (YTH domain-containing protein 3), ZC3H13 (a zinc finger protein), and WTAP (Wilms' tumor 1-associating protein) were significantly different in myocardial infarction tissues, demonstrating the key role of N6-methyladenosine (m6A) regulator in myocardial infarction. WTAP was significantly reduced in the MI group, and maintaining the normal expression of WTAP may be a preventive or ameliorative means for the treatment of MI. In addition, three subtypes with different clinical characteristics were constructed based on the expression profile of m6A regulatory factor, suggesting that they are potential therapeutic strategies for patients with myocardial infarction, which may provide new clues to identify biomarkers for clinical treatment and diagnosis.

In addition, significant progress has recently been made in the direct reprogramming of non-muscle cells into functional CM *in vitro* and *in vivo* through the forced expression of cardiac reprogramming factors. For cardiac reprogramming, the common initiating cell population, cardiac fibroblasts, is largely heterogeneous in its molecular characteristics and functional state, and the cell fate transition process is not synchronized, making it difficult to further elucidate this reprogramming process using conventional bulk genomic techniques. ScRNA-seq helps illuminate the underlying mechanisms of cardiac reprogramming. Liu et al. (57) analyzed changes in gene expression during cardiac programming based on scRNA-seq data and found enrichment of factors involved in mRNA processing and splicing. This study found that Ptbp1 depletion enhanced the transformation of fibroblasts to CM, ultimately improving reprogramming efficiency. Stone et al. (58) also investigated the transcriptional dynamics of reprogramming in mouse cardiac fibroblasts using scRNA-seq. In the future, single-cell multiomics will provide more comprehensive insights into gene regulatory networks and provide a theoretical basis for better applications of cardiac reprogramming.

Fibroblasts transform into an inflammatory phenotype immediately after myocardial infarction. During proliferation, fibroblasts are angiogenic, responsible for the formation of new extracellular matrix and down-regulation of angiogenesis. Cardiac fibroblasts are very suitable as key effector cells for the treatment of infarction due to their abundance, heterogeneity, phenotypic plasticity, and ability to secrete a variety of inflammatory mediators and repair growth factors. By understanding the regulatory factors of each state of fibroblasts and determining the role of fibroblasts in different stages of cardiac remodeling, it is of great significance to understand the biological effects of damaged and remodeled cardiac fibroblasts for the improvement of myocardial infarction by targeting fibroblasts.

## Angiogenesis

The heart contains an extremely heterogeneous network of microvessels. However, knowledge of this network and its impact on disease is limited (59). Blood supply interruption caused by MI is a key determinant of infarct size and subsequent deterioration in cardiac function. Recent studies demonstrated that functional angiogenesis in the infarcted area may delay disease progression and improve outcome (60, 61). Therefore, heart repair should be enhanced by restoring the vascular network. Su et al. used scRNA-seq to identify the transcriptional phenotypes of premature venous cells in developing mouse heart undergoing vein-to-artery transition independent of coronary blood flow (62). This experiment showed that cardiac angiogenesis is related both to embryonic heart development and cardiopathology. Single-cell sequencing of tissues from healthy and infarcted mouse hearts showed that the transcription factor zinc finger e-box binding homeobox 2 (ZEB2) was upregulated in stressed cardiomyocytes, induced cardioprotective cross-talk between cardiomyocytes and endothelial cells, and enhanced post-ischemic angiogenesis (15). Myocellular-specific ZEB2 downregulation leads to impaired myocardial contractility and post-infarction healing. By contrast, myocellular-specific ZEB2 overexpression improves myocardial cell survival and cardiac function by stimulating angiogenesis and improving cardiac repair. Therefore, ZEB2 is a promising angiogenic signaling mediator in response to cardiomyocyte injury. In neonatal mouse hearts, arterial endothelial cells migrate from the capillaries to the infarcted areas and form collateral arteries vital to heart regeneration (63). In adult mice, preexisting endothelial cells form new coronary vessels after injury (64). Single-cell RNA sequencing was used to explore endothelial cell dynamics and individual post-MI cellular responses. Endothelial cell proliferation was associated with changes in metabolic gene expression (16). A trajectory analysis showed that 3–7 d post-MI, most endothelial cells entered a transient state characterized by mesenchymal gene expression and metabolic adaptation. They, then, returned to baseline at 14 d post-MI and did not remain in a long-term mesenchymal state. Mesenchymal activation may promote endothelial cell migration and clonal expansion and regenerate vascular networks (23). Multi-spectral pedigree tracking was combined with single-cell RNA sequencing to study the origin, proliferation dynamics, and transcriptional profiles of new angiogenic endothelial cells in adult mouse hearts. Ten transcriptional heterogeneous endothelial subsets were defined, and they may enhance angiogenesis and tissue regeneration after ischemic injury. Resident EC subsets with progenitor cell-like functional properties contributed to new blood vessel formation 7 d post-MI. Serous vesicle-associated protein (Plvap) demonstrated endothelial specificity and was upregulated in both ischemic mouse and human hearts (23). Plvap regulated human endothelial proliferation *in vitro* and could promote endogenous neovascularization in infarcted myocardium (65). This study provides high-resolution information about the hierarchical transcriptional status of endothelial clonal expansion, which could be used to develop therapeutic targets promoting new blood vessel formation post-MI and potentially improving prognosis. It also furnishes evidence for the clonal expansion of endothelial cells, but not for the possibility that bone marrow cells or EMT participate in neovascularization (65). This discrepancy might partially account for the use of autologous cells. Adaptive angiogenesis occurs in response to myocardial ischemia and hypoxia injury. While *de novo* angiogenesis is inherently restorative in this situation, it may become maladaptive if is not modified and remodeled correctly. Therefore, it is essential to clarify revascularization to predict and treat MI effectively.

## Discussion

Myocardial infarction involves sudden cardiomyocyte death and is accompanied by white blood cell and lymphocyte infiltration from adjacent blood vessels to clear necrotic debris. It is followed by the replacement of repair cells such as myofibroblasts, endothelial cells, and anti-inflammatory (M2) macrophages. Myofibroblasts and endothelial cells are intricately involved in intercellular communication and play key roles in heart remodeling. It is important to understand the cellular proinflammatory and repair mechanisms that occur during heart remodeling. Over the past decade, numerous efforts have been made to clarify the genetic and functional diversity of heart cells. Defining heart cell interactions is a sound strategy for increase our understanding of the normal and the diseased heart. Cell lineage tracing, flow cytometry, and batch RNA sequencing analyze cell diversity based on specific cell type markers. However, recent advent of the single-cell RNA sequencing technology has enabled the exploration of individual cell diversity. It has redefined cardiac cell subpopulations and identified related cell subpopulations and new cell types through their cell-specific transcriptomic characteristics. These findings are changing our understanding of cell composition and may help identify potential therapeutic targets for various heart diseases (50). Therefore, a better understanding of post-stress heart change is essential to develop focused, targeted, and effective therapies.

Cellular heterogeneity is a moderate variation in the intercellular transcriptome caused by the surrounding microenvironment, and scRNA-seq can effectively analyze it (66). One of the first studies applying scRNA-seq to healthy and diseased hearts intended to observe heterogeneity within the same heart cell type. Heterogeneity was detected in the cardiomyocyte population. Non-pathological cardiomyocytes showed a significant gradient in the expression levels of cardiac markers such as actin α myocardial 1 and α-myosin heavy chain. Different transition stages can be defined and their hierarchy can be inferred by assigning each cell to a pseudo-time locus. Pseudo-time analyses provide clues regarding the earliest cellular events that drive cells toward a particular lineage. Researchers can predict the pedigree trajectory of individual cells on the basis of single-cell transcriptomic data. A combination of pedigree tracing and single-cell transcriptomic analysis will help elucidate disease progression (47). Ni et al. (47) use pseudo-time trajectory and Chromatin immunoprecipitation sequencing (ChIP-Seq) analyses identified Rel as a key factor inducing macrophage differentiation in MI. Trajectories obtained *via* quasi-temporal analysis are not supplemented by real differentiation time points and only furnish suggestions for the putative differentiation mechanism. Hence, experimental validation is required (24). Single-cell sequencing has revealed a key cardiac fibroblast subpopulation that plays a pathogenic role in MI progression (67). A hallmark of the progression from MI to heart failure is excessive ECM accumulation which, over time, impairs systolic and diastolic heart function and increases the risks of arrhythmia and death. Therefore, targeting cardiac fibroblast activation is a novel approach toward reducing interstitial fibrosis and improving post-MI remodeling. Single-cell sequencing data have identified multiple fibroblast subtypes and their post-MI activation. Myofibroblasts are the primary expression form (22, 41). Numerous genes specifically expressed in fibroblasts were identified and could serve as cellular markers. These include Ddah1 and cytoskeleton-related protein 4 (CKAP4) (34).

After cardiac ischemia, angiogenesis forms new capillaries from preexisting capillary beds and involves endothelial cell proliferation, germination, and migration. Angiogenesis is highly controlled, depends on the balance between pro-angiogenic and anti-angiogenic factors, and results from complex interactions among growth factors, endothelial cells, pericytes, fibroblasts, smooth muscle cells, and the ECM (35). Multi-chromatographic tracing and single-cell transcriptomics were used to characterize the cellular dynamics of vascular endothelial regeneration and revealed that this process is driven by various differentiated endothelial cell populations. Endothelial regeneration requires the activation of stress response genes such as Atf3. Blood vessels defective in their repair capacity express relatively low levels of Atf3. The foregoing findings provide important insights into the cellular dynamics and mechanisms driving the responses to vascular injury.

Single-cell sequencing can be used to study communication among cardiac cell types. Characterizing the cardiac cell interaction network comprising the heart is necessary to understand cardiac homeostasis and disease (68). In 2018, Skelly performed scRNA-seq on the non-cardiomyocyte portion of a mouse heart (69). Based on previous research, a list of potential ligand-receptor pairs was constructed and applied to mouse scRNA-seq data. There was a dense intercellular communication network among all cardiac cell types. The most communicative were fibroblasts. Most cells express tens to hundreds of ligands and receptors and pair them to create intricate signaling networks. Nevertheless, the effects of these interactions on target gene expression in receptor cells are not fully understood (66). Browaeys et al. (70) recently developed the NicheNet tool that models single cell-level intercellular communication by linking ligands to target genes. This tool models the mechanisms by which gene expression in one cell is affected by interactions with another. It also indicates the types of signaling mediators involved. Single cell analysis has also been used to pair ligands with their corresponding receptors in specific cell types and infer the interactions that might influence cell behavior.

## Limitations

There are certain technical problems associated with the current scRNA-seq methods. Traditional flux limitation, restrictions in determining the appropriate cell number and sequencing depth, and data processing impediments (71) are important challenges associated with scRNA-seq. Most single-cell sequencing models are, in fact, merely simple MI models. Clinically speaking, however, MI is the result of myocardial blood flow interruption caused by coronary artery occlusion. Hence, its underlying pathogenesis is, in fact, coronary atherosclerosis. Therefore, future studies should establish a model of MI based on an atherosclerosis model. The lack of functional validation in most of the foregoing studies underscores a technical obstacle, namely, the combination of high-dimensional transcriptome sequencing data with protein expression and fibroblast structure and function measurements. As scRNA-seq technology evolves, it is expected that the afore-mentioned technical issues will be rectified, and future scRNA-seq methods will generate valuable data for cardiovascular research. Single-cell transcriptome sequencing is performed at the transcriptional level, however, transcriptional levels are poorly correlated with protein levels. The future will still need to incorporate other techniques to better elucidate the dynamics of health and disease states.

## Summary

Single-cell technology offers unprecedented opportunities to systematically uncover cellular heterogeneity and dynamic molecular events during tissue development and disease progression. The clinical medicine of cardiovascular disease may benefit from this technological advance in a number of ways. First, the study of dynamic cellular changes during disease initiation and progression may reveal new diagnostic biomarkers, which may have great potential for immunosurveillance and clinical diagnosis of cardiovascular disease in the future. Second, single-cell techniques are useful in identifying novel therapeutic targets that might be obscured by conventional analytical methods. Using single-cell sequencing can detect disease-specific cell subsets or cell interactions that are critical to disease pathogenesis, provide new insights into the treatment of drug resistance, or help identify molecular targets for drug resistance, from which new therapies can be designed to improve treatment sensitivity. Finally, the application of single-cell sequencing may help to trace the genetic origins of diseases, while proteomics may help to classify disease phenotypes to facilitate the development of precision medicine. With these tools, single-cell approaches promise to update our understanding of cardiovascular biology and disease and advance clinical precision medicine and decision making for the foreseeable future.

## Author Contributions

LL, MW, and QM designed the article structure. LL, YL, and MW wrote the manuscript. LL and QM helped map the figures and revise the manuscript. GS, JY, and XS were responsible for the supervision and project administration. All authors discussed, edited, and approved the final version.

## Funding

This work was supported by the National Natural Science Foundation of China (Grant no. 81973514), the Key Laboratory project of Chinese Academy of Medical Sciences: New Drug discovery based on classic prescriptions (Grant no. 2018PT35030), and the Guangdong Provincial key field research and development plan project (Grant no. 2020B111111002).

## Conflict of Interest

The authors declare that the research was conducted in the absence of any commercial or financial relationships that could be construed as a potential conflict of interest.

## Publisher's Note

All claims expressed in this article are solely those of the authors and do not necessarily represent those of their affiliated organizations, or those of the publisher, the editors and the reviewers. Any product that may be evaluated in this article, or claim that may be made by its manufacturer, is not guaranteed or endorsed by the publisher.
